# Obstetric Sutures Gone Astray: A Bladder Erosion of a Cerclage Suture Leading to Recurrent Urinary Tract Infections

**DOI:** 10.7759/cureus.101097

**Published:** 2026-01-08

**Authors:** Fatima Hamid, Ksenia Basargin, Noor Farghaly, Erum Azhar, Ali Azadi

**Affiliations:** 1 Obstetrics and Gynecology, Creighton University School of Medicine, Phoenix, USA; 2 Obstetrics and Gynecology Residency Program, Creighton University East Valley Arizona, Gilbert, USA; 3 Obstetrics and Gynecology, Arizona College of Osteopathic Medicine, Midwestern University, Glendale, USA; 4 Urogynecology, Creighton University School of Medicine, Phoenix, USA

**Keywords:** bladder erosion, cervical cerclage, complicated uti, cystoscopy, luts, mcdonald cerclage, recurrent uti

## Abstract

Cervical cerclage is generally considered a safe procedure; however, rare complications, such as suture erosion and migration into adjacent organs, can occur. We present a case of a 31-year-old multiparous female who presented with a three-year history of recurrent urinary tract infections (UTIs), dysuria, urinary frequency, intermittent gross hematuria, and pelvic pain. She had a history of two prior cervical cerclages. Multiple courses of antibiotics failed to resolve her symptoms. CT imaging revealed a curvilinear hyperdense foreign body within the bladder. A cystoscopy was performed. Intraoperatively, a calcified Prolene suture - presumed to be a remnant from a prior cerclage - was identified at the bladder base and excised entirely. The patient had an uneventful postoperative course, with a follow-up CT confirming complete removal of the suture. The patient remained asymptomatic, with no recurrence of UTIs or hematuria on her follow-up visits.

This case highlights the importance of considering suture erosion in patients with recurrent urinary symptoms, especially in women with a history of pelvic procedures. Early recognition and definitive surgical removal can provide complete resolution of patient symptoms and optimize patient outcomes.

## Introduction

Cervical insufficiency (CI) is a pregnancy complication characterized by painless cervical dilation and shortening, which can lead to second-trimester pregnancy loss or preterm delivery [1]. Management involves placement of a cervical cerclage through a surgical procedure in which a suture is placed circumferentially around the cervix [2]. The purpose of a cerclage is to reinforce cervical tensile strength and maintain closure of the cervix to prevent premature deliveries and miscarriages [3]. During the second trimester, cervical length is assessed between 18 and 24 weeks of gestational age, due to the prevalence of CI, which ranges between 0.1% and 2% [4]. In women with previous spontaneous preterm birth, single gestations, and cervical length less than 25 mm, cerclage significantly prevents preterm birth as well as composite perinatal mortality and morbidity [5]. Cervical cerclages have demonstrated a reduced risk of preterm births (39% to 49%) in women who were clinically diagnosed with cervical shortening via transvaginal ultrasound or based on a history of preterm birth [5,6]. 

Cerclages have been beneficial in prolonging the gestational period and thus have improved obstetrical outcomes; however, they can be associated with complications such as membrane rupture, urinary tract infections (UTIs), hematuria, cervical tear, uterine rupture, and erosion of adjacent organs [3]. Iatrogenic injury to the bladder is a rare complication that can occur during the passage of the suture through the anterior lip of the cervix, as well as with the anterior placement of the knot [1]. This injury could be attributed to the initial suture placed on the cervix, which may directly damage the bladder wall or erode over time, leading to knot migration [7]. 

We present a case of a 31-year-old gravida 2, para 1-1-0-2 (one term delivery, one preterm delivery, and two living children) female, who presented with recurrent UTIs and was ultimately found to have a bladder erosion from a cervical cerclage suture.

## Case presentation

A 31-year-old female, gravida 2, para 1-1-0-2, presented to the urogynecology clinic with a three-year history of intermittent dysuria, urinary frequency, intermittent gross hematuria, and pelvic pain, which worsened with sitting and prolonged walking. She reported worsening of her symptoms over the past six months, which significantly impacted her quality of life.

Her past surgical history was notable for a loop electrosurgical excision procedure (LEEP) and two prior cervical cerclages. The first cerclage was placed five years prior to her initial visit and was removed in-office prior to vaginal delivery at 36 weeks of gestation. The second cerclage was placed using the McDonald technique at 12 weeks of gestation and removed at vaginal delivery at 38 weeks of gestation.

Her physical examination was unremarkable. Initial urinalysis results showed +1 leukocytes, and urine cultures were positive for *Enterococcus faecalis*. She completed a course of amoxicillin and potassium clavulanate 500 mg/125 mg, followed by subsequent courses of sulfamethoxazole/trimethoprim 800 mg/160 mg; however, her symptoms did not resolve. A repeat urinalysis 11 days later showed +1 leukocytes, 2+ nitrates, and 3+ blood. An outpatient cystoscopy was attempted but was limited due to the patient’s discomfort and poor visualization.

**Figure 1:**
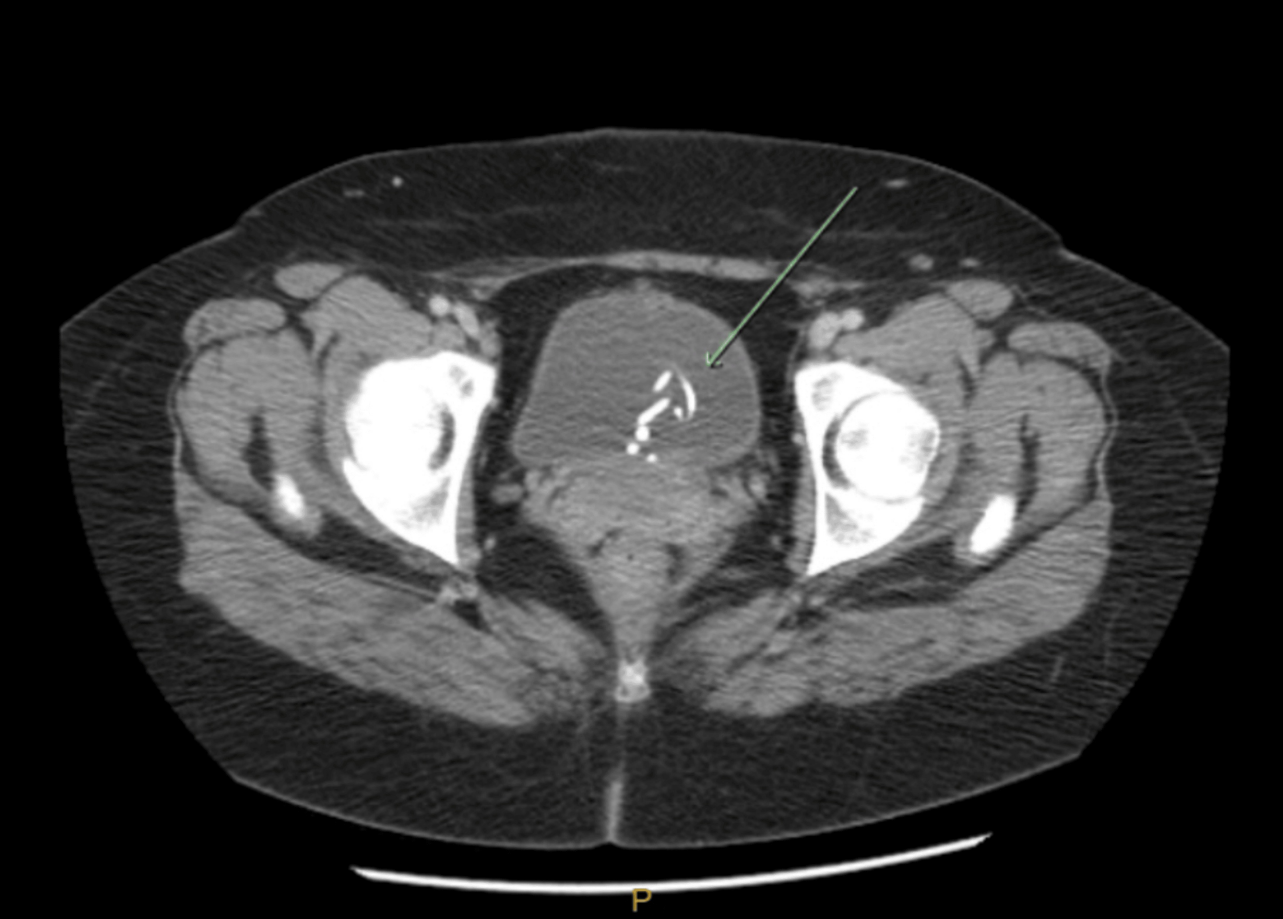
CT scan of the abdomen and pelvis showing a fragmented, curvilinear foreign object in the bladder (green arrow).

A non-contrast CT scan showed two to three curvilinear, fragmented foreign bodies in the bladder (Figure 1). Given the above findings, the patient was admitted to the hospital for further evaluation by cystoscopy under anesthesia.

**Figure 2:**
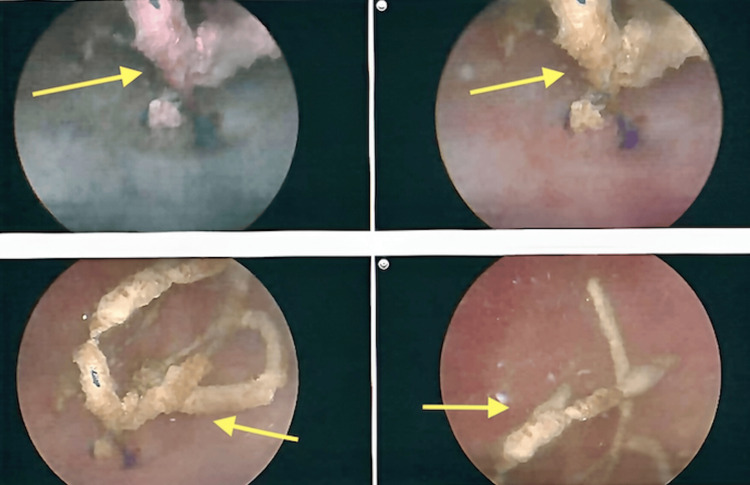
Cystoscopy showing calcified Prolene suture at the bladder base (yellow arrows).

The patient underwent an examination under anesthesia with concurrent hysteroscopy and cystoscopy. Hysteroscopy demonstrated a normal cervix and endometrial cavity. Cystoscopy showed a calcified object at the bladder base, which was completely excised endoscopically using cold scissors (Figure 2). The excised material consisted of blue Prolene suture fragments, consistent with her prior cerclage. A Foley catheter, with continuous bladder irrigation, was placed postoperatively to reduce fistula risk. The pathology report confirmed the presence of multiple fragments of Prolene suture with calcification. The postoperative CT scan confirmed the complete removal of the foreign bodies. 

The postoperative course was uncomplicated. Urinalysis and urine culture were negative on postoperative day 6. The patient reported complete resolution of urinary symptoms and pelvic pain on the follow-up visit in the office.

## Discussion

Cervical cerclage is an essential intervention in the management of CI; however, it carries inherent risks. Most complications are seen in the immediate postoperative period and include rupture of membranes, chorioamnionitis, cervical lacerations, and suture displacement [3]. A systematic review by Alani et al. [8] revealed that the most commonly reported long-term complication of cervical cerclage was fistula formation. These delayed complications can occur from as early as 12 weeks to as late as 13 years after cerclage placement. In our case report, the patient became symptomatic within one year but remained undiagnosed for three years, thus highlighting the diagnostic challenges associated with these late-term complications.

Although intravesical foreign bodies are typically associated with iatrogenic injuries during pelvic or urologic surgeries, misplacement or erosion of a cervical suture into the bladder is a rare but important complication [9,10]. The clinical presentation mimics symptoms of UTIs, such as dysuria, urinary frequency, urgency, and suprapubic or urethral pain [9,10]. This often leads to a diagnostic delay. In this case report, the patient’s urinary symptoms were ultimately attributed to an eroded suture visualized on cystoscopy. Intravesical foreign bodies serve as a nidus for infection and calculus formation [9,10], which explains the patient’s recurrent UTIs.

Other long-term complications associated with cerclage include genitourinary fistulas. A retrospective study on patients with cerclage-related genitourinary fistulas by Massengill et al. showed that 83.3% had a prior history of at least one cervical surgery [7]. Our patient had a history of LEEP and two cervical cerclages, which may have predisposed her to delayed suture migration and erosion. The McDonald technique was the most common approach for cerclage placement, seen in 81.8% of the cases in the Massengill series [7], and was the technique used in our patient. There was also an association seen between the McDonald method and late-term complications, although this may reflect its widespread use rather than causality.

Meticulous technique is required to prevent erosion and migration of the suture. Ruan et al. [11] described a patient with a 10-year history of recurrent urinary symptoms and retained fragments of cerclage, despite multiple attempts at removal of suture fragments. This emphasized that inadequate visualization during extraction can cause chronic morbidity. In our case presentation, the cerclage was removed when the patient presented in early labor. Although no immediate complications were documented, the emergent nature of cerclage removal in our patient’s case may have contributed to incomplete extraction, which subsequently led to migration and erosion of the suture into the bladder wall.

The type of suture material used for cerclage can also influence the risk of migration and erosion. Monofilament and braided sutures are commonly used for cerclage placement. Monofilament sutures are preferred due to a lower risk of infection, but braided sutures are known to provide greater tensile strength. However, the largest randomized controlled trial showed no significant difference in adverse outcomes between the two suture types, and it is important to note that monofilament sutures were associated with a higher incidence of complications related to cerclage removal, which included incomplete removal and the need for anesthesia [12]. In this case report, a Prolene monofilament suture was used for the second cervical cerclage. It is possible that the suture’s durability and stiffness play a role in migration into the bladder wall. Evidence-based data on the ideal suture material for cerclage are lacking.

Patient morbidity can be reduced by early recognition of suture exposure or migration. There should be a high index of suspicion in patients who present with recurrent urinary symptoms, especially those who have a history of pelvic surgery or cerclage placement. In these patients, there should be a low threshold to perform a cystoscopy, as direct visualization can identify foreign bodies in the bladder. Failure to identify foreign bodies in the bladder can cause recurrent infection, stone formation, or fistula formation.

Suture erosion into the bladder is a rare but important late complication of cervical cerclage. This case emphasizes consideration of these long-term complications in patients with a history of cerclage placement, who present with recurrent urinary symptoms without an identifiable cause. These complications can be prevented by complete extraction of the suture under direct visualization and by carefully reviewing the operative notes. Clinicians should consider cystoscopy in all patients with unexplained urinary symptoms, with a history of pelvic surgery or cerclage, to help prompt diagnosis and ensure optimal patient outcomes.

## Conclusions

Bladder erosion of a cerclage suture is an exceedingly rare cause of recurrent UTIs. This case highlights the importance of imaging and cystoscopy in women with unexplained urinary symptoms, especially those who have a history of prior gynecologic procedures. Definitive treatment is by surgical removal. Injury to the bladder can be prevented by careful anatomical consideration and meticulous suture placement. It is important to ensure complete suture removal at the time of cerclage extraction to minimize the risk of suture erosion. Clinicians should maintain a high index of suspicion for iatrogenic foreign bodies to facilitate timely diagnosis and optimal patient outcomes.
